# Treatment-resistant Bipolar Disorder and Thyroid Cancer

**DOI:** 10.1192/j.eurpsy.2022.1048

**Published:** 2022-09-01

**Authors:** H. Jemli, U. Ouali, A. Aissa, Y. Zgueb, R. Jomli

**Affiliations:** 1 University of tunis elmanar, Faculty Of Medicine Of Tunis, manouba, Tunisia; 2 Razi Hospital, Psychiatry A, manouba, Tunisia

**Keywords:** bipolar disorders, thyroid function, resistance, Anxiety disorders

## Abstract

**Introduction:**

Bipolar disorder (BD) is a chronic and recurrent illness frequently associated with functional deterioration and treatment challenges. High rates of thyroid dysfunction have been found in patients with BD, compared to the general population.

**Objectives:**

To illustrate through a case-report the therapeutic challenges of treatment-resistant bipolar disorder and its relationship with thyroid dysfunction.

**Methods:**

Case report of a 41-year-old male patient with BD and comorbid anxiety disorders who has been diagnosed with thyroid cancer and underwent total thyroidectomy.

**Results:**

Mr B is a 41 year old patient diagnosed with BD and comorbid anxiety disorders (panic disorder, social anxiety disorder and generalized anxiety disorder) at age 18. He has presented in total 17 relapses and was hospitalized 7 times between the ages of 18 and 24. He experienced predominantly major depressive episodes with mixed features and debilitating anxiety symptoms. He was put on several treatments including a combination of mood stabilizers, antidepressants and benzodiazepines. Due to unsatisfactory treatment response, he was put on clozapine 150mg to 175mg/d combined with valproic acid, clonazepam. In 2009, the patient developed a nodular goiter caused by papillary thyroid carcinoma and underwent total thyroidectomy and radioactive iodine therapy. Following the surgical operation and stabilization of thyroid functioning, a decrease in the number of relapses and the severity of mood and anxiety symptoms have been noted.

**Conclusions:**

This case reports highlights the importance of thyroid function assessment in patients with bipolar disorder and the possible correlation to treatment resistance and symptom severity.

**Disclosure:**

No significant relationships.

